# A Forger of Diplomas Caught

**Published:** 1870-02

**Authors:** 


A Forger of Diplomas Caught.—We find the following in the Toledo, Ohio, *Commercial*, of a recent date:

In the latter part of October last, Dr. Alexander McMillen, of Genoa, Ottawa county, received a letter, dated Lucas, Richland county, Ohio, in which the writer proposed to furnish the Doctor with a diploma from the "American University, of Philadelphia," provided Dr. McMillen would pay two hundred dollars for the same. The Doctor at once consulted his attorney, who advised him to answer the letter and lead the Lucas doctor, who signed himself "G. Galloway, M. D.," into a more full exposure of the business, if possible. In the first letter the Genoa doctor was asked to obtain the certificates of two physicians that he had been for five years a successful practicing physician, but in subsequent letters it was said this might be dispensed with, and he would get the diploma without such certificate. Dr. McMillen, finding that he could get nothing further out of Dr. Galloway, ordered a diploma, which was to be forwarded with charges to the amount of $200, "C. O. D." He also stated that he would probably visit the agent for these diplomas, and intimated that he had a friend who would like a diploma.

The correspondence ended about the first inst., and a few days ago the diploma arrived at Genoa, and purported to hail from No. 225 North Twelfth street, Philadelphia. A warrant was at once issued, and on Friday the constable at Genoa started down to Richland county after his man. Lucas is a small village about seven miles from Mansfield, and the constable went to the latter place and inquired for Dr. Galloway, who lives at Lucas. No one knew such a man, but soon he saw a person who pointed to a man across the street, and remarked that the person pointed out resided at Lucas, and that his name was Dr. Burch. The constable went to the man and asked if he knew a physician at Lucas by the name of Galloway. "I answer to that name," said he; "Is this Dr. McMillen?" "No," replied the constable; "but you are my prisoner." The man was at once taken to Genoa, without being allowed to return home. On Saturday he was taken before a justice at Genoa, but he got his case continued until the 12th inst., he being sent to jail at Port Clinton to await the time of trial.

